# Supplementary material for: Pulmonary vasodilator use in very preterm infants in United States children’s hospitals
Source: J Perinatol. 2025 May 2;45(10):1382–8. doi: 10.1038/s41372-025-02309-x (PMC12479341; doi:10.1038/s41372-025-02309-x)
Supplement: Supplementary file 1 — Supplementary Figure 1 [file 41372_2025_2309_MOESM1_ESM.docx]

**Supplementary Figure S1.** Cohort composition flowchart

Excluded (n=5534)

- CHD other than PDA, ASD, or VSD (n=4627)
- CDH and/or lung hypoplasia/aplasia (n=571)
- Chromosomal abnormalities (n=336)

42962 very preterm subjects

Excluded (n=426854)

- Admitted to hospital with > 50% missing data for gestational age (n=77821)
- Gestational age not between 22-31 weeks (n=343606)
  - Missing GA (n=43420)
  - GA outside range (n=300186)
- Gestational age discrete variable and diagnosis code category discordant (n=5427)

Excluded (n=11046)

- Admission length of stay < 7 days (n=5005)
- Died prior to 36 weeks postmenstrual age (n=2262)
- Discharged alive on respiratory support prior to 36 weeks postmenstrual age (n=1373)
- Discharged alive and off respiratory support before 28 weeks postmenstrual age (n=37)
- Admitted after 36 weeks postmenstrual age (n=2369)

54008 very preterm subjects

480862 subjects admitted to a PHIS participating hospital NICU between 2011-2021

37428 very preterm subjects
