# Supplementary material for: Pulmonary vasodilator use in very preterm infants in United States children’s hospitals
Source: J Perinatol. 2025 May 2;45(10):1382–8. doi: 10.1038/s41372-025-02309-x (PMC12479341; doi:10.1038/s41372-025-02309-x)
Supplement: Supplementary file 2 — Supplementary Table 1 [file 41372_2025_2309_MOESM2_ESM.docx]

**Supplementary Table S1.** ICD-9 and -10 codes considered for cohort exclusion criteria

| **Congenital heart diseases**  ICD9  745.11 Double outlet right ventricle  745.2 TOF  745.69 Other endocardial cushion defects  746.00 Congenital pulmonary valve anomaly, unspecified  746.02 Stenosis of pulmonary valve, congenital  746.09 Other congenital anomalies of pulmonary valve  746.1 Tricuspid atresia and stenosis, congenital  746.2 Ebstein’s anomaly  746.3 Congenital stenosis of aortic valve  746.4 Congenital insufficiency of aortic valve  746.5 Congenital mitral stenosis  746.6 Congenital mitral insufficiency  746.81 Subaortic stenosis, congenital  746.82 Cor triatrium  746.83 Infundibular pulmonic stenosis, congenital  746.84 Obstructive anomalies of heart, congenital, not elsewhere classified  746.85 Coronary artery anomaly, congenital  746.87 Malposition of heart and cardiac apex  746.89 Other specified congenital anomalies of heart  746.9 Unspecified congenital anomaly of heart  747.10 Coarctation of aorta (preductal) (postductal)  747.21 Congenital anomalies of aortic arch  747.29 Other congenital anomalies of aorta  747.31 Pulmonary artery coarctation and atresia  747.39 Other anomalies of pulmonary artery and pulmonary circulation  747.40 Anomaly of great veins, congenital, unspecified  747.49 Other anomalies of great veins  747.89 other unspecified congenital anomaly of circulatory system  ICD10  Q201 Double outlet right ventricle  Q203 Discordant ventriculoarterial connection  Q204 Double inlet ventricle  Q205 Discordant atrioventricular connection  Q208 Other congenital malformations of cardiac chambers and connections  Q209 Congenital malformation of cardiac chambers and connections, unspecified  Q212 Atrioventricular septal defect  Q219 Congenital malformation of cardiac septum, unspecified  Q221 Congenital pulmonary valve stenosis  Q222 Congenital pulmonary valve insufficiency  Q223 Other congenital malformations of pulmonary valve  Q225 Ebstein’s anomaly  Q226 Hypoplastic right heart syndrome  Q228 Other congenital malformations of tricuspid valve  Q229 Congenital malformations of tricuspid valve, unspecified  Q230 Congenital stenosis of aortic valve  Q231 Congenital insufficiency of aortic valve  Q232 Congenital mitral stenosis  Q233 Congenital mitral insufficiency  Q238 Other congenital malformations of aortic and mitral valves  Q240 Dextrocardia  Q241 Levocardia  Q242 Cor triatrium  Q243 Pulmonary infundibular stenosis  Q245 Malformation of coronary vessels  Q246 Congenital heart block  Q248 Other specified congenital malformations of heart  Q249 Congenital malformation of heart, unspecified  Q251Coarctation of aorta  Q254 Other congenital malformations of aorta  Q254.2 Hypoplasia of aorta  Q254.4 Congenital dilation of aorta  Q254.5 Double aortic arch  Q254.6 Tortuous aortic arch  Q254.7 Right aortic arch  Q254.9 Other congenital malformations of aorta  Q256 Stenosis of pulmonary artery  Q257.2 Congenital pulmonary arteriovenous malformation  Q257.9 Other congenital malformations of pulmonary artery  Q258 Other congenital malformations of other great arteries  Q259 Congenital malformation of great arteries, unspecified  Q261 Persistent left superior vena cava  Q262 Total anomalous pulmonary venous connection  Q263 Partial anomalous pulmonary venous connection  Q264 Anomalous pulmonary venous connection, unspecified  Q268 Other congenital malformations of great veins  Q288 Other specified congenital malformations of circulatory system  **Chromosomal anomalies**  ICD9  279.11 DiGeorge syndrome  758.0 Down’s  758.1 Patau’s  758.2 Edwards’  758.32 Velo-cardio-facial syndrome (DiGeorge)  75833 Other microdeletions  75839 Other autosomal deletions  7585 Other conditions due to chromosomal anomalies  758.6 Gonadal dysgenesis  758.81Other conditions due to sex chromosome anomalies  758.89 Other conditions due to chromosome anomalies  758.9 Conditions due to anomaly of unspecified chromosome  ICD10  D821 DiGeorge syndrome  Q902 Tristomy 21, translocation  Q909 Down syndrome, unspecified  Q912 Trisomy 18, translocation  Q916 Trisomy 13, translocation  Q922 Partial trisomy  Q925 Duplications with other complex rearrangements  Q927 Triploidy and polyploidy  Q928 Other specified trisomies and partial trisomies of autosomes  Q929 Trisomy and partial trisomy of autosomes, unspecified  Q934 Deletion of short of chromosome 5  Q935 Other deletions of part of a chromosome  Q9381 Velo-cardio-facial syndrome  Q9382 Williams syndrome  Q9388 Other microdeletions  Q9389 Other deletions from the autosomes  Q962 Karyotype 46, X with abnormal sex chromosome, except iso (Xq)  Q998 Other specified chromosomal abnormality  Q999 Chromosomal abnormality, unspecified  **Congenital diaphragmatic hernia and/or lung hypoplasia/aplasia**  ICD9  519.4 Disorders of diaphragm  553.3 Diaphragmatic hernia without mention of obstruction or gangrene  748.5 Agenesis, hypoplasia, and dysplasia of lung, congenital  756.6 Anomalies of diaphragm  ICD10  J986 Disorders of diaphragm  K440 Diaphragmatic hernia with obstruction, without gangrene  K449 Diaphragmatic hernia without obstruction or gangrene  Q336 Congenital hypoplasia/dysplasia of lung  Q339 Congenital malformation of lung, unspecified  Q790 Congenital diaphragmatic hernia |
| --- |
