# Supplementary material for: Pulmonary vasodilator use in very preterm infants in United States children’s hospitals
Source: J Perinatol. 2025 May 2;45(10):1382–8. doi: 10.1038/s41372-025-02309-x (PMC12479341; doi:10.1038/s41372-025-02309-x)
Supplement: Supplementary file 3 — Supplementary Table 2 [file 41372_2025_2309_MOESM3_ESM.docx]

**Supplementary Table S2.** CPT, ICD-9 and ICD-10 codes considered for defining study variables

| **CPT codes for PDA occlusion or ligation**  02LR0CT Occlusion of Ductus Arteriosus with Extraluminal Device, Open Approach | |
| --- | --- |
| 02LR0DT | Occlusion of Ductus Arteriosus with Intraluminal Device, Open Approach |
| 02LR0ZT | Occlusion of Ductus Arteriosus, Open Approach |
| 02LR3CT | Occlusion of Ductus Arteriosus with Extraluminal Device, Percutaneous Approach |
| 02LR3DT | Occlusion of Ductus Arteriosus with Intraluminal Device, Percutaneous Approach |
| 02LR3ZT | Occlusion of Ductus Arteriosus, Percutaneous Approach |
| 02LR4CT | Occlusion of Ductus Arteriosus with Extraluminal Device, Percutaneous Endoscopic Approach |
| 02LR4DT | Occlusion of Ductus Arteriosus with Intraluminal Device, Percutaneous Endoscopic Approach |
| 02LR4ZT | Occlusion of Ductus Arteriosus, Percutaneous Endoscopic Approach |
| 02VR0CT | Restriction of Ductus Arteriosus with Extraluminal Device, Open Approach |
| 02VR0ZT | Restriction of Ductus Arteriosus, Open Approach |
| 02VR3CT | Restriction of Ductus Arteriosus with Extraluminal Device, Percutaneous Approach |
| 02VR3DT | Restriction of Ductus Arteriosus with Intraluminal Device, Percutaneous Approach |
| 02VR3ZT | Restriction of Ductus Arteriosus, Percutaneous Approach |
| 02VR4DT | Restriction of Ductus Arteriosus with Intraluminal Device, Percutaneous Endoscopic Approach |
| **ICD codes for VSD** | |
| ICD9 | 745.4 |
| ICD10 | Q21.0 |
| **ICD codes for ASD** | |
| ICD9 | 745.5 |
|  | 745.61 |
| ICD10 | Q21.1 |
| **ICD codes for grade 3 and 4 IVH** | |
| ICD9 | 431 – Intracerebral Hemorrhage |
|  | 77213 – Intraventricular hemorrhage, Grade III |
|  | 77214 – Intraventricular hemorrhage, Grade IV |
| ICD10 | I611 – Nontraumatic intracerebral hemorrhage in hemisphere, cortical |
|  | I614 – Nontraumatic intracerebral hemorrhage in cerebellum |
|  | P5221 – Intraventricular (nontraumatic) hemorrhage, grade 3, of newborn |
|  | P5222 - Intraventricular (nontraumatic) hemorrhage, grade 4, of newborn |
|  | P524 – Intracerebral (nontraumatic) hemorrhage of the newborn |
|  | P526 – Cerebellar (nontraumatic) and posterior fossa hemorrhage of newborn |
| **ICD codes for stage II and III NEC** | |
| ICD9 | 5672 – Other suppurative peritonitis |
|  | 5679 – Unspecified peritonitis |
|  | 5680 – Peritoneal adhesions (postoperative) (postinfection) |
|  | 7776 – Perinatal intestinal perforation |
|  | 56983 – Perforation of intestine |
|  | 77752 – Stage IInecrotizing enterocolitis in newborn |
|  | 77753 – Stage III necrotizing enterocolitis in newborn |
| ICD10 | K5532 – Stage 2 necrotizing enterocolitis |
|  | K5533 – Stage 3 necrotizing enterocolitis |
|  | K631 – Perforation of intestine (nontraumatic) |
|  | P772 – Stage 2 necrotizing enterocolitis in newborn |
|  | P773 – Stage 3 necrotizing enterocolitis in newborn |
|  | P780 – Perinatal intestinal perforation |
